# Electrospinning based biomaterials for biomimetic fabrication, bioactive protein delivery and wound regenerative repair

**DOI:** 10.1093/rb/rbae139

**Published:** 2024-12-03

**Authors:** Xinyi Dai, Wei Nie, Hua Shen, Hans-Günther Machens, Kai Böker, Shahed Taheri, Wolfgang Lehmann, Yi Shen, Arndt F Schilling

**Affiliations:** Department of Plastic and Reconstructive Surgery, Shanghai Ninth People’s Hospital, Shanghai Jiao Tong University School of Medicine, Shanghai 200011, China; Wake Forest Institute for Regenerative Medicine, Wake Forest School of Medicine, Winston Salem, NC 27103, USA; Department of Plastic and Reconstructive Surgery, Shanghai First People’s Hospital, Shanghai Jiao Tong University School of Medicine, Shanghai 200080, China; Department of Plastic and Hand Surgery, Klinikum Rechts der Isar, Faculty of Medicine, Technical University of Munich, Munich 81675, Germany; Department of Trauma Surgery, Orthopaedic Surgery and Plastic Surgery, University Medical Center Göttingen, University of Göttingen, Göttingen 37075, Germany; Department of Trauma Surgery, Orthopaedic Surgery and Plastic Surgery, University Medical Center Göttingen, University of Göttingen, Göttingen 37075, Germany; Department of Trauma Surgery, Orthopaedic Surgery and Plastic Surgery, University Medical Center Göttingen, University of Göttingen, Göttingen 37075, Germany; School of Biomedical Engineering, Shanghai Jiao Tong University, Shanghai 200240, China; Department of Trauma Surgery, Orthopaedic Surgery and Plastic Surgery, University Medical Center Göttingen, University of Göttingen, Göttingen 37075, Germany

**Keywords:** electrospinning, nanofibrous matrices, biomimetic scaffold, protein delivery, wound healing

## Abstract

Electrospinning is a remarkably straightforward and adaptable technique that can be employed to process an array of synthetic and natural materials, resulting in the production of nanoscale fibers. It has emerged as a novel technique for biomedical applications and has gained increasing popularity in the research community in recent times. In the context of tissue repair and tissue engineering, there is a growing tendency toward the integration of biomimetic scaffolds and bioactive macromolecules, particularly proteins and growth factors. The design of ‘smart’ systems provides not merely physical support, but also microenvironmental cues that can guide regenerative tissue repair. Electrospun nanofibrous matrices are regarded as a highly promising tool in this area, as they can serve as both an extracellular matrix (ECM)-mimicking scaffold and a vehicle for the delivery of bioactive proteins. Their highly porous architecture and high surface-to-volume ratio facilitate the loading of drugs and mass transfer. By employing a judicious selection of materials and processing techniques, there is considerable flexibility in efficiently customizing nanofiber architecture and incorporating bioactive proteins. This article presents a review of the strategies employed for the structural modification and protein delivery of electrospun nanofibrous materials, with a focus on the objective of achieving a tailored tissue response. The article goes on to discuss the challenges currently facing the field and to suggest future research directions.

## Graphical Abstract

**Figure:**
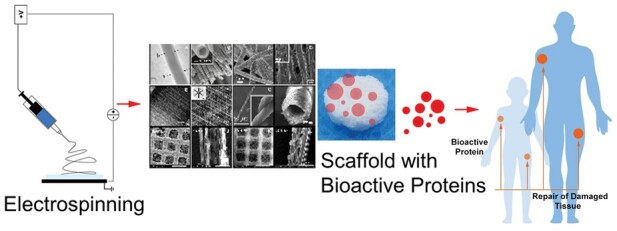

## Introduction

Electrospinning is a notable fiber-fabrication technique to produce non-woven fibrous materials with typical fiber diameters in the order of a few micrometers down to tens of nanometers [1]. In 1900, it was first patented by Cooley [2, 3], but only truly surfaced as a utilizable technology for spinning small-diameter fibers in 1934, when Formhals patented a process and an apparatus using electric charges to spin synthetic fibers for the textile industry and organic polymer science [4]. Recently the unique advantages of generating nanoscale scaffolds with controlled surface morphology have gained increasing attention for applications in drug delivery and tissue regeneration [5, 6].

Tissue regenerative repair, such as wound regenerative healing, is one of the holy grails of clinical therapy, which has evolved from medical science and has been a rapidly growing interdisciplinary research field that applies the principles of engineering and life science, which covers the fundamentals of combining cells, scaffolds and bioactive molecules to assemble functional substitutes or release promoting substances to achieve successful tissue regeneration. Initially, drug containing dressings or pre-cultured cells and synthetic scaffold complexes were developed for implantation into a defect area to enhance the expected cell proliferation and biological structure formation [7–9]. Due to different problems with cell harvest, maintenance and implantation, recently there is an increasing trend to shift the tissue engineering strategy to the development of acellular scaffolds [10]. These can be easily stored and are therefore immediately available for implantation at an injured site. They are designed to motivate natural regenerative processes through both structural support and signaling induced by biomolecules released in a controlled manner from extracellular matrix (ECM)-mimicking matrices.

The ECM is composed of over 300 biologic materials, including collagens, proteoglycans and glycoproteins [11]. Collagens are the main structural proteins of the extracellular space and provide up to 35% of the whole body protein content [12, 13]. They consist of triple-helices, forming elongated fibrils. Depending on the degree of mineralization the surrounding tissue may be compliant (tendon), rigid (bone) or rigid to compliant (cartilage) [13]. Another main component of ECM is proteoglycans. These molecules are heavily glycosylated by adding glycosaminoglycans (GAGs) chains to the core protein complex [14]. Glycoproteins on the other hand are core proteins modified by oligosaccharide chains (glycans) attached to nitrogen (N-glycosylation) or oxygen (O-glycosylation) [15]. Cells are constantly changing and rebuilding ECM (i.e. synthesis, degradation and reassembling) through chemical modifications of components, such as phosphorylation or glycosylation [16].

The ECM is a major component of the cellular microenvironment and involved in many cellular processes like differentiation, proliferation, cell death and cancer development [16]. Consequently, modulation or reproduction of ECM components is a possible way to modify these cellular processes. Such modulation can be used to favor improved regenerative processes, like the recruitment of progenitor cells toward the defect area with subsequently improved repair of the damaged tissue [17]. To engineer such interactive scaffolds as ‘smart’ systems for *in vivo* use, fiber size, morphology and hierarchical structured assemblies can be controlled to elicit specific responses from cells and tissues [18, 19]. Electrospun fibrous and porous structures that simulate natural ECM architecture could open new possibilities here. The right spatiotemporal combination of growth factors and cytokines has always been considered a prerequisite for the control of cellular components, which are essential for tissue repair [20]. Therefore, especially the possibility of controlled release of signaling molecules from electrospun materials after implantation to activate desired physiological responses is inspiring. Furthermore, electrospinning gives the engineer a better control over the properties compared to other fiber forming techniques or drug delivery systems (e.g. polymer micelles, liposomes, hydrogels, nano/microspheres and cast films) [21].

In this article, we review the strategies used to incorporate bioactive molecules especially growth factors into electrospun nanofibrous matrices. We report techniques used to control fibrous structures for the regeneration of specific target tissues, discuss existing challenges and suggest future directions and potential applications.

## Basic principles of electrospinning and factors related

Electrospinning is a very simple and straightforward method to change flow properties of polymer solutions. It allows the production of continuous nanofibers through an electrically charged jet [22]. So far, a large number of polymers as well as modifications of electrospinning appliances have been tested to endow electrospinning with specific features and tailor the structure of resultant fibers for tissue regeneration.

**Figure 1.**
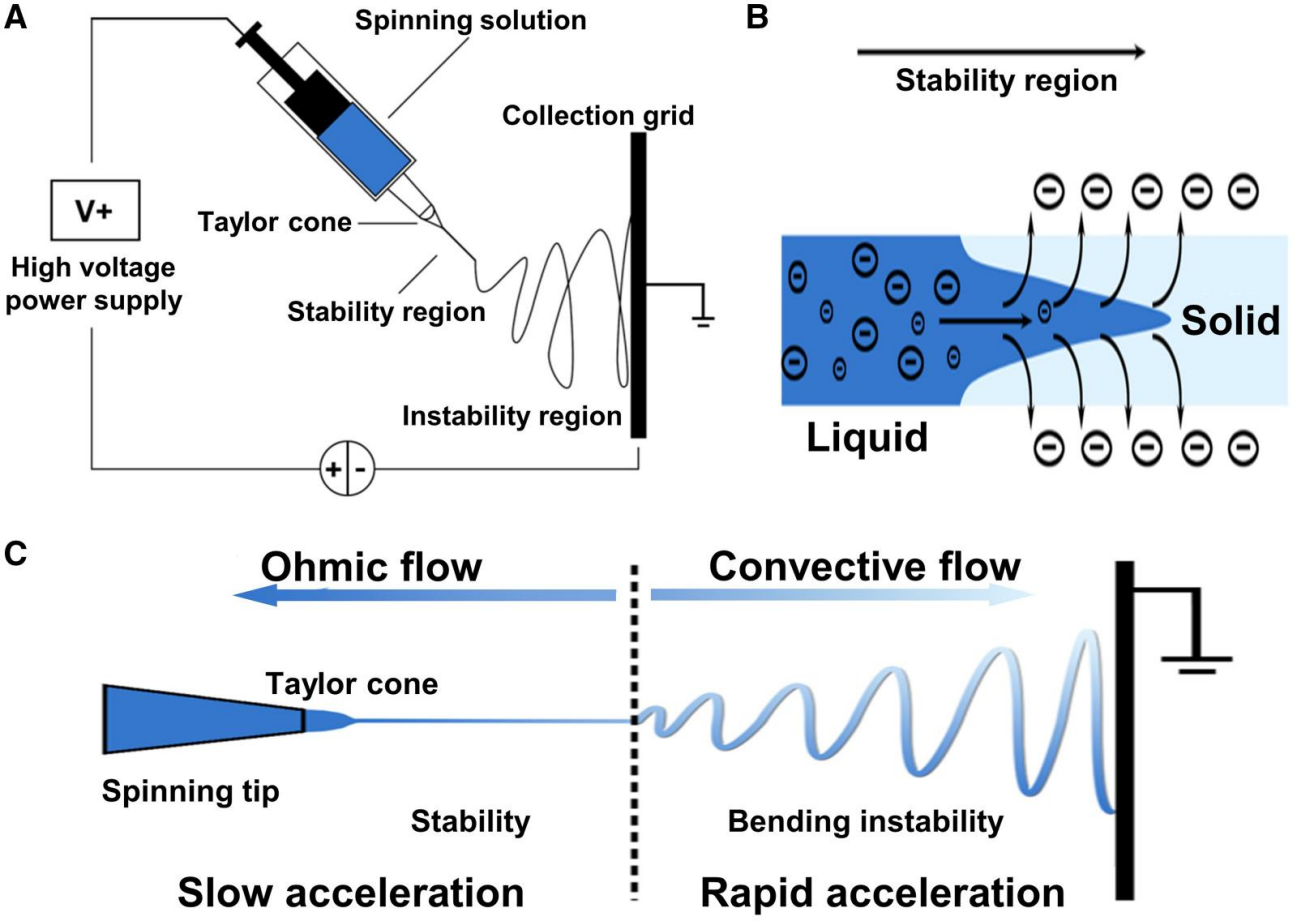
Schematic illustration of conventional and modified electrospinning technique. (**A**) Basic setup of electrospinning. (**B**) Solution jet dries in flight and the charge migrates to the surface of the jet. (**C**) As the mode of current flow changes from ohmic to convective, electrically driven chaotic bending instability occurs with the resulting fiber diameter down to a few nanometers.

During the electrospinning process, the polymer solution (or melt) in the syringe pump will be injected at a constant feed-rate to the nozzle. If a high voltage is applied, the pendant drop of polymer solution will be charged and the hemispherical surface of the droplet elongates to form a conical object known as Taylor cone. When the voltage further increases and surpasses a threshold value to overcome the surface tension of the droplet, a charged liquid jet is ejected from the tip of the Taylor cone toward the earthed collector (Figure 1A). As the jet dries in flight due to solvent evaporation, the mode of current flow changes from ohmic to convective because the charge migrates to the surface of the fiber (Figure 1B). This leads to elongation and thinning of the jet. Then the motion of segments of the jet grows rapidly into an electrically driven chaotic bending instability and a whipping process initiates small bends in the fiber. This is probably due to repulsive interactions between like-charged liquids in the jet [23], ending with increasing transit time and path length until the jet is finally deposited on the grounded collector [24]. This process finally leads to generation of dry polymer fibers with nanometer-scale diameters (Figure 1C).

To gain a deeper understanding of the electrospinning process, it is essential to investigate the formation of the Taylor cone, the impact of solution properties and other influencing factors. The Taylor cone represents a fundamental phenomenon in the context of electrospinning, occurring when a high-voltage electric field is applied to a droplet of polymer solution or melt at the tip of a needle or capillary. The formation of the Taylor cone is a consequence of the introduction of a polymer solution into a capillary, whereby surface tension causes the solution to form a droplet at the tip of the needle. The application of an electric field between the needle and a grounded collector results in the exertion of electrostatic forces on the charged liquid within the droplet. In the initial stages, the surface tension exerts a dominant influence, maintaining the spherical shape of the droplet. As the electric field strength increases, the electrostatic forces exerted on the charged liquid in the droplet cause the surface tension to be overcome, resulting in the elongation of the droplet into a cone shape, known as the Taylor cone. At a critical voltage, when the electrostatic forces exceed the surface tension, a charged jet is ejected from the Taylor cone. This marks the beginning of the fiber formation process in electrospinning. The formation of the Taylor cone is influenced by a number of factors, which subsequently affect the characteristics of the resulting fibers. These include the viscosity of the solution, the surface tension and the voltage, among others. A solution of high viscosity will exhibit greater resistance to deformation, necessitating the application of a higher voltage in order to initiate the formation of the Taylor cone. Additionally, higher surface tension will impede cone formation, whereas lower surface tension will facilitate jet initiation. It is also essential to note that an optimal voltage is necessary for stable cone formation. If the voltage is insufficient, no jet will form, whereas if it is excessive, the jet may become unstable. The impact of electrospinning voltage on fiber diameter is inconsistent, with some studies indicating a reduction in diameter at higher voltages, while others report an initial increase followed by a decrease [25, 26].

In addition to the formation of the Taylor cone, the properties of the spinning solution exert a significant influence on the electrospinning process, including factors such as conductivity, viscosity and concentration. The electrical conductivity of the polymer solution is a critical parameter that exerts a significant influence on the electrospinning process. The ability of the solution to carry charges under the applied electric field is a determining factor in fiber formation, jet stability and fiber morphology. An elevated electrical conductivity augments the number of charges borne by the jet, thereby intensifying the stretching of the jet due to Coulombic repulsion between like charges, which in turn produces a reduction in fiber diameter. Consequently, a higher conductivity frequently yields thinner fibers, as the augmented charge density within the jet enables more effective stretching. Conversely, low-conductivity solutions may result in the formation of thicker fibers or even beads due to insufficient stretching. While high conductivity facilitates stretching, excessively high conductivity can also induce instabilities, resulting in branching or the ejection of multiple jets from the Taylor cone. This may result in a loss of control over the uniformity of the fibers. In many cases, salts or ionic compounds are added to the solution in order to increase conductivity. The selection and dosage of these additives can be used to precisely control the diameter of the resulting fibers. For example, the addition of a small amount of salt to a polymer solution often results in a notable reduction in fiber diameter. The optimal range of conductivity depends on the specific polymer type and solution viscosity. In the case of high-viscosity solutions, higher conductivity is typically required to generate the necessary charges for electrospinning. It is essential to maintain a balance of conductivity to ensure that the jet remains stable and the fibers are smooth and continuous.

Technically, there are also several other parameters that greatly affect fiber formation. These include operational conditions such as applied voltage, polymer flow rate, capillary-collector distance and ambient parameters like temperature, humidity and air velocity have also been found to be able to influence the morphology and structure of the resulting fibers [27]. In short, fiber diameter is mainly influenced by the concentration and viscosity of the polymer solution, applied voltage and collecting distance. Meanwhile, the porosity and pore sizes of the resulting nanofibrous scaffolds can be further controlled by changing fiber diameter and their packing density [28]. Appropriate adjustment of all or some of these parameters optimizes the resulting electrospun fiber structure toward desired morphologies and structural properties. Based on the analysis of these influencing factors, some technical modifications have also been used to improve the performance or production efficiency of electrospun fibers to meet certain special applications, especially in biomedicine, such as side-by-side electrospinning [29, 30], co-axial electrospinning [31, 32], multi-jet electrospinning [33], needleless (bubble) electrospinning [34] and so on.

## Modulating therapeutic effects by engineering of nanofibers

The therapeutic effects of the fabricated nanofibrous scaffolds can be to some extent controlled by a meticulous selection of suitable materials and production methods. The requirements of this engineering are mainly set by the purpose of application in the specific type of tissue. For a successful incorporation and delivery of bioactive proteins, additional attention must be paid to select appropriate materials to ensure adequate cellular affinity as well as full protection of the encapsulated biomacromolecules against denaturation and inactivation. All this is further determined by process ability of the material and electrospinning technique being adopted. If all can be successfully combined, this results in nanofibers with tailored mechanical and topographical cues that fit for specific tissue regeneration requirements [35, 36].

### Material selection

**Figure 2.**
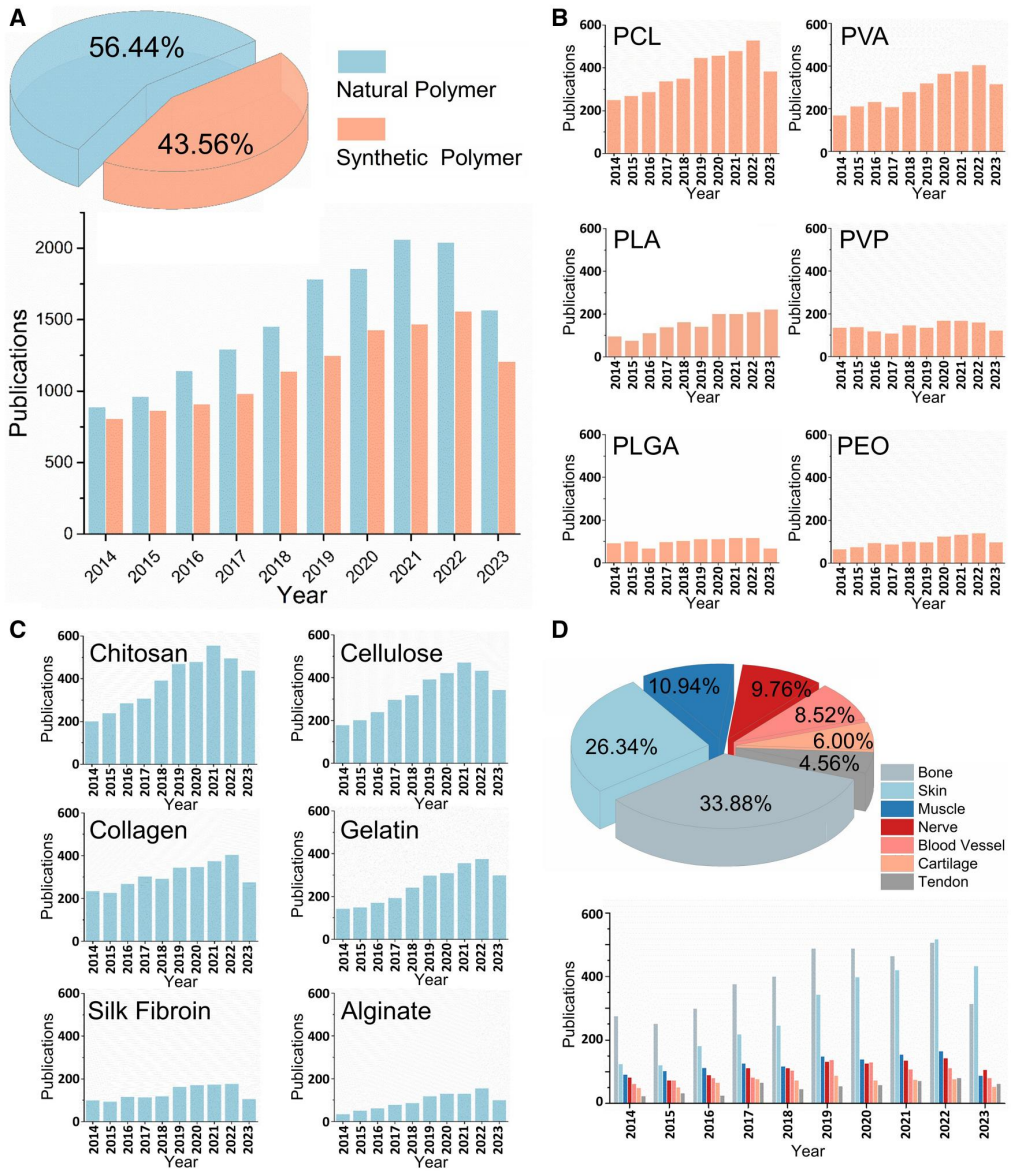
Publications trends for synthetic and natural polymers processed by electrospinning from January 2014 to November 2023. (**A**) Number of research papers and patents published in the last 10 years (synthetic polymers vs. natural polymers). (**B**) Publication trends for commonly used synthetic polymers for electrospinning. (**C**) Publication trends for commonly used natural polymers for electrospinning. (**D**) Publication trends for different tissue types repaired by electrospun scaffolds. Sources: ISI – Web of Science (https://clarivate.com/products/web-of-science/).

As in all applications for tissue regeneration and drug delivery, the selected material and its degradation products should have no toxicity and should not induce immune responses. There is a wide range of material choices to prepare electrospun nanofibrous scaffolds for tissue engineering applications depending on the nature and structure of a particular tissue to be regenerated. Electrospinning has been successfully applied for processing of more than 200 synthetic and natural polymers, ceramics and their composites and even metals [37, 38]. A concise overview of recent publication trends, which encompass research papers and patents, utilizing synthetic and natural polymers in electrospinning-based studies is illustrated in Figure 2A. The subsequent analysis delves into detailed information on the most commonly adopted synthetic and natural polymers.

#### Synthetic polymers

Synthetic polymers offer significant advantages over natural polymers in tissue regeneration applications, particularly when employed in electrospinning processes. Their well-defined molecular structure, controllable molecular weight and predictable behavior during electrospinning make them highly desirable for tailoring mechanical, architectural and degradation properties.

Compared to natural polymers, synthetic polymers are minimally immunogenic, highly reproducible and cost-effective. Their predictability allows for precise customization of properties, facilitating their extensive use in tissue regeneration [39]. Among the widely used synthetic polymers are linear aliphatic polyesters such as hydrophobic poly(L-lactic acid) (PLA), poly(ε-caprolactone) (PCL) and poly(lactic-co-glycolic acid) (PLGA). Additionally, water-soluble polymers like polyvinyl alcohol (PVA), poly(ethylene oxide) (PEO), poly(vinyl pyrrolidone) (PVP) and poly(ethylene glycol) (PEG) find applications in tissue engineering.

As an example, PVA is a hydrophilic biodegradable and eco-friendly polymer with good chemical and thermal stability. It has been shown to be nontoxic, biocompatible, non-carcinogenic and mechanically durable. Therefore, electrospun PVA is widely used for wound dressings, implants and artificial organs [40], such as bone [41, 42], skin [43] or nervous tissue [44]. However, due to its instability in water, it is unsuitable for drug delivery [45]. To overcome this, cross-linked PVA nanofibers have gained significant attention in the field of drug delivery due to their unique properties and versatile applications [46, 47]. Similarly, PEO is readily soluble in most aqueous solutions and is a biocompatible, porous material with very low toxicity. It is often used as fiber forming material because it can be electrospun without defects from aqueous solutions in a rather narrow range of conditions and therefore has become an excellent candidate for scaffolding for tissue regeneration (e.g. cartilage [48]) or wound dressing [49]. In a study conducted by Liu et al., electrospun nanofibrous scaffolds were prepared using a blend of PEO, chitosan and kaolin. The results demonstrated that the scaffolds facilitated accelerated healing of back wounds in rats, with a minimal inflammatory response observed within 14 days [50]. PGA is another synthetic thermoplast polymer with many favorable properties including biocompatibility, bioabsorbability, moderate degree of crystallinity, high melting point but low solubility and tensile strength [51]. Due to its hydrophilic nature, monofilaments can be relatively quickly degraded *in vivo*, and electrospun PGA has been considered as an attractive candidate for tissue engineering scaffolds or structures with controlled drug release [52, 53]. PLA, a synthetic non-immunogenic and nontoxic polymer extracted from cornstarch, is a biocompatible material with excellent biodegradability, resorbability, mechanical strength and good solubility in organic solvents [54]. PLA degrades slower than PGA due to the differences of the hydrophobic methyl groups of the backbone [55]. It can serve as tissue engineering scaffold, such as polymeric extracellular matrix used in the regeneration of myocardial tissues [56] or hard tissues like bone [57]. However, the hydrophobic surface of PLA limits its application in a hydrophilic bio-environment. In this case, hydrophilic polymers [58, 59] are used to improve hydrophilicity. They have been shown to improve cell growth on the material [60, 61]. PCL is a biodegradable semi-crystalline polymer with a low melting point (*Tm*) and good resorption capacity. This low-cost material also has a high thermal stability and high mechanical strength and is generally considered to be nontoxic and tissue-compatible [62]. Moreover, the degradation rate of PCL is much slower than PGA or PLA due to its hydrophobicity and its by-products do not cause an acidic environment [63]. Consequently, it has already been used for the production of nanofibrous biomaterials through electrospinning for a wide range of biomedical applications such as controlled drug delivery [64], wound dressing [65, 66], ‘long-lasting’ absorbable sutures [67], 3D scaffolds for tissue engineering of cartilage [68], bone [69], ligaments [70], tendons [71], blood vessels [72] and even breast tissue [73]. The general trends of publications about synthetic polymers processed by electrospinning for tissue regeneration purpose in the past 10 years is summarized in Figure 2B.

Although synthetic polymers offer versatility, challenges associated with these nanofibers include process scalability, control over fiber morphology, inferior mechanical properties and considerations of biodegradability and biocompatibility. Ongoing research aims to address these limitations through advancements in material science and process engineering. Recent research aims to overcome these limitations, with notable contributions including works by Li et al. on advanced electrospun nanofiber materials for tissue engineering [74], Wei et al. on scalable electrospinning techniques [75] and Zhang et al. on biodegradable electrospun nanofiber scaffolds for drug delivery [76]. Some polymers, due to inherent hydrophobicity, lack cell affinity and active sites for cell recognition and binding. Modifications may be necessary to overcome these limitations. Additionally, during degradation, acidic by-products produced by certain polymers can impact the local biological environment, potentially leading to prolonged inflammatory responses.

#### Natural polymers

In contrast to synthetic polymers, natural polymers usually contain specific molecular domains to support and guide cell development so that the biological interaction between material and the host tissue is enhanced [77]. By emulating the natural ECM, it is feasible to attain not only exceptional biocompatibility and biodegradability, but also a substrate for cell adhesion and migration, which is pivotal for the regeneration of layered structure tissue such as skin. On the other hand, natural polymers most often lack mechanical properties and may lose their strength and dimensional stability due to gelation and rapid hydrolysis [78]. Furthermore, their processing properties are relatively poor, when compared to their synthetic counterparts [79]. Therefore, additional steps are often needed to improve their mechanical properties, water resistance as well as biodegradation rate for tailored tissue regeneration [80].

Nanofibers from several natural ECM proteins have been previously reported, representing attractive alternatives for tissue regeneration applications. Collagen is the most abundant protein in mammals with favorable characteristics including resorbability, bio-degradability and non-inflammatority. This natural polymer usually contains three very long polypeptide chains that are twisted in triple helix regular shape to provide strength and elasticity to tissues like tendon or skin. Matrices from electrospun collagen (type I [81], type II [82], type III [83, 84] or its derivatives, e.g. gelatin [85]) have been shown to offer close mimicry of native ECM for cell adhesion, infiltration and proliferation. They therefore have been widely investigated for the regeneration of a wide variety of tissues like cartilage [84], bone [86], skin [87] and blood vessels [88] as well as for development of favorable drug delivery systems [89]. On the other hand, this approach is relatively expensive and the spun fibers do not yet match the mechanical strength of the natural polymer. Meanwhile, if the constituents are extracted from animal tissue, there is a risk of immunogenicity [90]. Fibrinogen, whose molecule contains six chains and two arginine–glycine–aspartate (RGD) integrin-binding sites [91], has a proven track record of tissue engineering due to its innate ability of inducing cellular interaction and subsequent scaffold remodeling [92]. As a naturally occurring soluble protein responsible for blood coagulation, it is capable of forming precursors (fibrin) of fibrous structures (clot) through reaction with thrombin. This acts as natural provisional matrix for the healing process [93], it is easily degradable, non-immunogenic and promotes cell migration [94]. Therefore, fibrinogen was studied as a possible component for fabrication of biomimicking fibrous constructs with mechanical properties similar to those of native tissue upon electrospinning. Furthermore, fibrinogen based micro and nanostructures have been proven to be effective vehicles to consecutively encapsulate and deliver multiple growth factors and proteins (e.g. VEGF, EGF, NGF, BMP, TGF, FGF, FN [95, 96]) to assist specific tissue regeneration (e.g. blood vessel, skin, nerve and bone) [96]. Silk fibroin has a favorable biocompatibility, adaptable biodegradability and good oxygen/water vapor permeability, which has recommended it for the use in a variety of 2D and 3D tissue engineering matrices. The natural polymer produced by spiders has a highly repetitive primary sequence that leads to significant homogeneity and high degree of crystallinity. This leads to unique mechanical properties [97], which make it particularly interesting for tissues which require a high tensile strength [98, 99], like skin [100], bone [101], cartilage [102] or ligament-like tissues [103]. Cellulose is one of the world’s most abundant natural biologic and renewable materials. It can be extracted from plants’ cell walls or obtained by growing bacteria colonies. It is a linear glucosic homopolymer with excellent biodegradability, biocompatibility and low cytotoxicity [104]. Inter- and intra-molecular hydrogen bonds among hydroxyl groups within the glucose units provide excellent mechanical properties to cellulose, whose electrospun fibers are far better than those of many commonly used electrospun materials applied in tissue engineering [105]. Interestingly, the stiffness, which mainly arises from hydrogen bonds, also represses its solubility. Cellulose derivatives with lower mechanical strength and susceptibility to rapid degradation have been designed. These materials however usually require post-modification processes if use in tissue regeneration applications is desired [106]. Even so, cellulose and its derivatives are generally accepted as promising materials for soft and hard tissue regeneration applications due to their economic advantages, easy production and accessibility as well as their tunable properties [107]. Through electrospinning, cellulose nanofibrous materials with geometric fiber arrangements similar to those of natural ECM can be produced [108]. This allowed to use them in a wider array of biomedical applications including drug delivery [109], wound dressing [110], tissue repair or healing replacement (e.g. skin [111], bone [112], dura-mater [113] and medical implants [114]). There are many other natural polymers commonly adapted for electrospinning to generate nanofibrous constructs for tissue regeneration applications, including chitin, chitosan [115], alginate [116] and hyaluronic acid (HA) [117]. The trends of publications about commonly used natural polymers for electrospinning in the past 10 years are summarized in Figure 2C.

As different polymers have their distinct set of advantages, it is reasonable to use polymer blends to increase the electrospinnability as well as to fabricate composite fibrous matrices as new materials with desirable properties. For example, nanofibers containing both synthetic polymer as backbone (strength and durability) and natural polymer (cell affinity) for cell attachment, exhibit not only suitable mechanical properties but also a bioactive surface [55]. Furthermore, the rate of biodegradation can be controlled by careful adjustment of the right components of the polymer alloy, to comply with the rate of new tissue formation. Such strategies have recently been reviewed by several groups [80, 118]. The mainly studied tissues for application of engineered electrospinning scaffolds within the last 10 years are summarized in Figure 2D.

### Structural control

Aside from biochemical signals, topographical cues of electrospun nanofibrous scaffolds are of equal importance to provide a favorable microenvironment for cell activity during regenerative processes [119]. Through careful selection of material and processing parameters, electrospinning has shown great flexibility in modulating the shape, size, morphology and structure of micro/nanostructures. This is advantageous for drug delivery systems, in particular the delivery of bioactive proteins. The elaborate engineering of scaffold’s morphology, porosity and composition is equally important in a recent trend of combining biomimetic scaffolds and growth factors to fabricate ‘smart’ bioactive systems capable of fine-tuning the release profile [120].

#### Morphology and size of nanofibers

Fiber morphology is mainly decided by resultant of three forces imposed on the liquid jet during electrospinning. As previously stated, electrostatic repulsion on the jet surface increases surface area, viscoelastic force primarily gives dimensional stability and surface smoothness to fibers, while surface tension tends to convert the liquid jet into spherical droplets [121]. Careful adjustment of these three forces dictates the morphology of the resulting fiber. Fiber diameter is crucial for various applications, influenced by polymer properties (e.g. molecular weight), solution properties (e.g. conductivity) and operating conditions (e.g. electric field strength, feeding rate) [122]. Higher viscosity results in thicker fibers, while more conductive solutions lead to smaller diameters [123, 124]. Incorporation of salt into the spinning solution is a frequently used method to increase its net charge density, and achieve a reduced fiber diameter [125]. There is an optimal feed-rate to achieve the narrowest fiber diameter [126], while increasing the feed-rate beyond that may produce ribbon-like fibers, wet fibers or even droplets [127, 128]. Standardization of experimental parameters is needed for a clearer understanding. Overall, careful adjustment of these factors is essential for controlling fiber morphology and diameter in electrospinning.

The morphology and size of electrospun nanofibers exert a considerable influence on their therapeutic efficacy. As evidenced in wound treatments, thin nanofibers offer a number of advantages, including a higher surface area to volume ratio (which typically signifies a greater capacity to absorb proteins), accelerated hydrolysis of the scaffold, enhanced collagen deposition and re-epithelialization, and the ability to emulate the native tissue environment [129, 130].

#### Secondary structure of nanofibers

**Figure 3.**
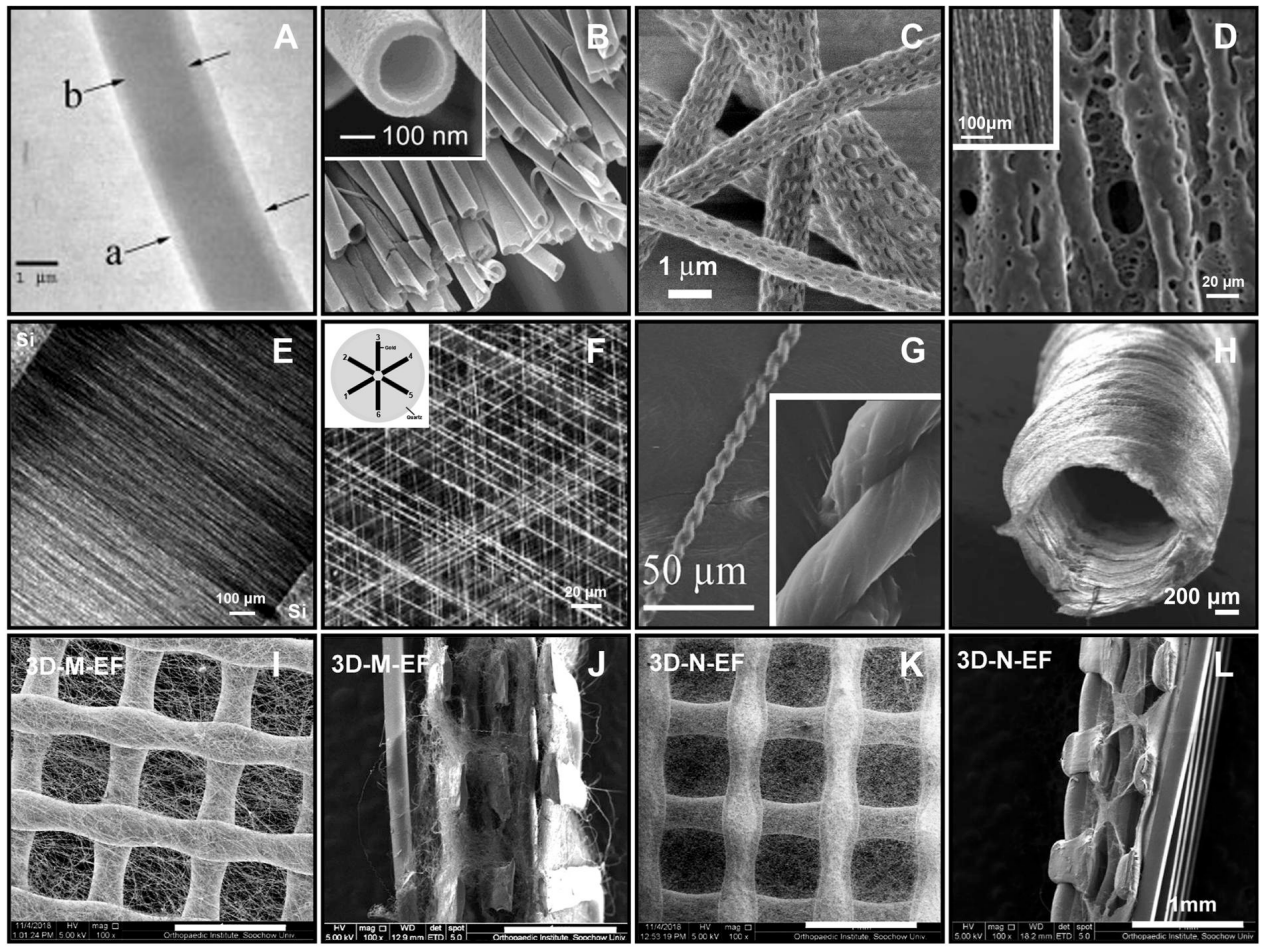
Structural control of electrospun nanofibers and their assembly for more complex architectures. (**A**) Core-sheath fiber. Reproduced with permission from [131]. Copyright 2004, John Wiley and Sons. (**B**) Hollow fiber. Reprinted with permission from [132]. Copyright 2004, American Chemical Society. (**C**) Porous fiber. Reproduced with permission from [133]. Copyright 2001, John Wiley and Sons. (**D**) Uniaxially aligned nanofiber obtained by rotating mandrel. Reproduced with permission from [134]. Copyright 2009, John Wiley and Sons. (**E**) Uniaxially aligned nanofiber produced by ‘gap technique’. Reprinted with permission from [135]. Copyright 2003, American Chemical Society. (**F**) Aligned multi-layer fiber with well-defined hierarchical structure. Reproduced with permission from [136]. Copyright 2004, John Wiley and Sons. (**G**) Electrospun fiber yarns. Reproduced with permission from [137]. Copyright 2010, John Wiley and Sons. (**H**) Tubular conduit structure. Reproduced with permission from [138]. Copyright 2008, Springer Nature. (**I**–**L**) Morphology of the 3D-printed scaffolds with electrospun microfibrous (3D-M-EF) and nanofibrous (3D-N-EF). Reproduced with permission from [139]. Copyright 2008, Elsevier.

Nanofibers with typical secondary structures, including core-sheath, hollow or porous fibers, can be fabricated through the process of electrospinning. Core-sheath structures can be successfully produced by co-axial (or sometimes emulsion) electrospinning (Figure 3A) [131, 132]. They are excellent candidates to exhibit features of both component materials, e.g. biocompatibility of natural polymers and mechanical strength of synthetic polymers. Moreover, core-sheath fibers demonstrate a sustained and controlled release of core-entrapped drugs. The core-sheath design helps maintaining the bioactivity of the drug thanks to the protection from the sheath [17], making them especially appealing for drug [140], protein and growth factor delivery [141]. Hollow nanofibers can be produced by direct co-axial electrospinning for specific applications [31, 142]. The core material is often dissolved with a selective solvent at the end of the process. For example, two viscous but immiscible liquids (oil and ethanol solution containing PVP and Ti(OiPr)_4_) were used to produce TiO_2_/PVP core-sheath nanofibers while the oil phase was selectively removed by solvent extraction (Figure 3B) [132]. Different substances can be embedded in hollow nanofibers to expand their applications [143].

The surface area of nanofibers can be increased tremendously when they become porous, which can accordingly improve cell attachment (Figure 3C) [133, 144]. Phase separation is the major mechanism used to obtain porous fibers, which is based on the separation of a thermodynamically unstable polymer solution into polymer rich (matrix) and polymer poor (pore) phases [145]. Solvent-rich regions begin to form when a more volatile solvent is used during the electrospinning process. When solvents evaporate, solvent-rich regions transform into pores [146]. Porous fibers of poly-L-lactide (PLLA) and polycarbonate (PC) can be directly prepared during electrospinning by the judicious selection of volatile solvents such as dichloromethane [133]. Another approach is to selectively remove a component from composite/blend nanofibers, such as the removal of one component through solvent dissolution while the other insoluble polymer remains intact [147]. From a biological point of view, inducing pore-like secondary structures to electrospun nanofibers may accommodate more nutrients and oxygen as well as the transportation of metabolic waste, which are essential for cell viability.

Thus, the presence of secondary structures of nanofiber can facilitate enhanced therapeutic efficacy in tissue regeneration, particularly in wound healing. This is achieved by increasing the surface area to volume ratio, which further strengthens protein integration and delivery. Additionally, it facilitates the regulation of biodegradable polymer degradation, which is crucial for drug delivery systems. Furthermore, the intricate nanotopographical features of scaffolds impart additional physical cues that influence cellular behavior [148]. This enables the mimicking of the ECM in terms of its physical structure. For further details on the utilization of these structures for bioactive molecule integration and delivery, please refer to Sections ‘Protein integration and delivery’, ‘Drug delivery and controlled release’, and ‘Regenerative scaffolds and ECM mimics’.

#### Alignment of nanofibers

The orientation of nanofibrous scaffolds is important to provide additional topographical cues for cellular activities [149]. Increased anisotropy in topography and structure affects both mechanical properties and cell activity (e.g. adhesion, proliferation, differentiation, alignment and migration) [150]. Evidence show that scaffolds made of aligned nanofibers are significantly stiffened in the direction of alignment, which is particularly helpful to mimic anisotropic load-bearing tissues like striated muscle, tendon or myocardium. The spatial orientation of nanofibers is controlled during electrospinning by altering jet movements *via* applying either mechanical, electrostatic or magnetic force. A rotating mandrel is commonly used as a modification of the electrospinning setup to convey mechanical force to the polymer jet and collect the resulting electrospun nanofibers as uniaxially aligned arrays [151]. Matthews et al. demonstrated that collagen fibrils electrospun onto a rotating mandrel (∼4500 rpm, linear velocity of mandrel’s surface > 1.4 m/s) yielded aligned deposition of fibrils along the axis of rotation. They concluded that through the control of fiber orientation, subtle mechanical properties can be tailored into the fabricated matrix [152]. Aligned electrospun nanofibers can also be produced by an electrostatic field-assisted assembly technique in which the mandrel is replaced by a tapered, wheel-like disk capable of collecting nanofibers in parallel arrays mostly at its tip-like edge [153]. Macroscopically aligned PCL fibers (rotating mandrel, 400–3000 rpm) coated with biocompatible fibrins are potential candidates for vessel regeneration (Figure 3D). They are reportedly able to enhance phenotype shift of human umbilical artery smooth muscle cells (HUASMCs) and maintain biological function of human umbilical vein endothelial cells (HUVECs) [134]. Setting an external electric field is another approach to manipulate and control the alignment of nanofibers. In a so-called ‘gap technique’, two conducting electrodes that are separated by a void gap are used to collect uniaxially aligned nanofibers across the gap (Figure 3E). This technique has the advantage of convenient transfer of the aligned fibers onto other solid substrates for further applications [135]. Likewise, an insulating substrate (e.g. quartz or polystyrene) with pair-wise metal electrodes patterned on the top has been established as the collector, through which both the position and the spatial orientation of the collected nanofibers can be readily controlled by varying the layout or configuration of the electrodes (Figure 3F) [136]. Well-defined hierarchical structures can be produced by sequential grounding of these pairs-wise electrodes [135]. Other patterns such as radially-aligned fibers [154] or microwell arrays [155] have been fabricated for wound closure and neural regeneration, respectively. Last but not least, aligned fibers can be fabricated under an external magnetic field if magnetic nanoparticles are mixed with polymers [156]. The so-called magnetic field-assisted electrospinning (MFAES), or magnetic electrospinning (MES) method, is shown to produce well-ordered arrays of nanofibers over large areas as thick matrix films [157].

#### Complex architecture of nanofibers (hierarchical structured assemblies)

Electrospinning stands as a versatile method for fabricating nanofibrous materials serving as scaffolds for tissue regeneration. Despite its efficacy, the small pore sizes inherent in electrospun scaffolds present challenges to cellular infiltration and hinder tissue regeneration. Addressing this issue demands diverse strategies, ranging from adjusting electrospinning parameters to employing post-electrospinning modifications. Increasing fiber diameters by adjusting polymer concentration or molecular weight enhances pore size in both synthetic and natural polymer scaffolds [158, 159]. Similarly, modifications in collector shape, such as using rotating mandrels or spherical dishes, have been explored [160, 161]. Utilizing wet baths as collectors with solvents like t-butyl alcohol (t-BuOH) enables adjustments in pore size and porosity [162]. Co-electrospinning sacrificial polymers or introducing salt particles followed by dissolution increases scaffold porosity [163, 164]. Additionally, chemical blowing agents induce pore formation during scaffold fabrication [165].

Beyond traditional electrospinning, techniques like depositing nanofibers on water or solid substrates followed by drawing, and applying shear/rotational force to create twisted nanofiber yarns (Figure 3G), offer avenues for complex scaffold construction [137, 166–168]. Textile-forming technologies such as braiding, weaving and knitting have been employed to mimic native tissues [169–174]. Layer-by-layer stacking allows tailoring scaffold properties by adjusting material properties and incorporating growth factors or drugs [175]. Combining fiber deposition with cell seeding enables the fabrication of cell-embedded multilayered architectures [176, 177]. Electrospinning facilitates the production of tubular conduits (Figure 3H) suitable for vascular or neural tissue engineering [138, 178]. By designing removable 3D collectors or simply rolling up electrospun nanofibrous mats, various conduit configurations can be achieved [179, 180]. For instance, hierarchical biomimetic scaffolds for bone regeneration can be created by rolling nanofibrous matrices concentrically [181].

While these strategies offer promising avenues for scaffold enhancement, each approach comes with its set of challenges and limitations. These may include difficulties in achieving uniform pore sizes, scalability issues and potential alterations in scaffold mechanical properties. A balanced understanding of these factors is crucial for informed scaffold design and application.

### Protein integration and delivery

In the context of clinical therapy, the incorporation of bioactive macromolecules for induced tissue regeneration into biomimetic nanofibrous scaffolds represents a crucial aspect, particularly in the case of wound healing [182]. Many of those biomolecules, like proteins are vulnerable to chemical (e.g. organic solvent) or physical processing (e.g. heating or electrical charges). Thus, it is not easy to retain their bioactivity and functional efficiency, both during and after electrospinning, and upon degradation after implantation. Preservation of the bioactivity of the incorporated therapeutic agents can be improved through careful material selection and adjustment of processing conditions, leading to desired encapsulation efficiencies [182]. Now, many improved procedures such as blending, co-axial processing, physical adsorption, covalent immobilization and microparticle incorporation have been investigated and proven to be effective for protein integration and delivery.

**Figure 4.**
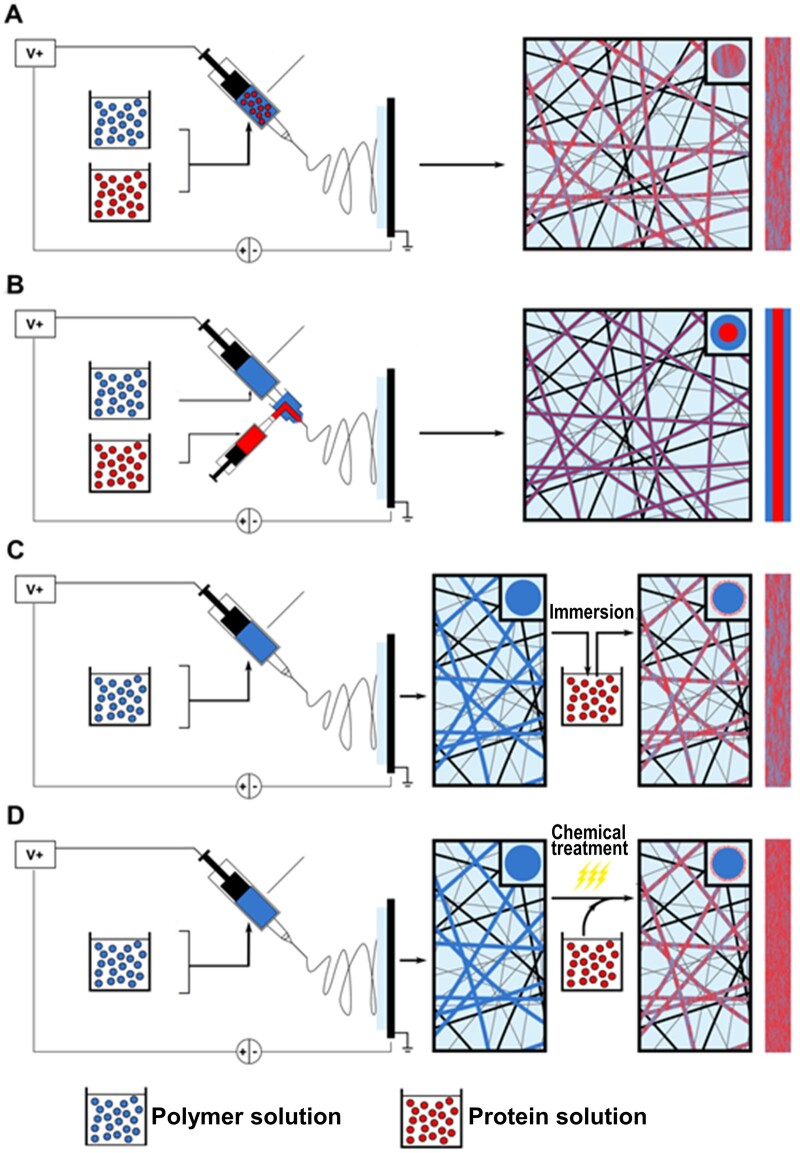
Schematic illustration of techniques used for bioactive protein integration and delivery. (**A**) Blend electrospinning, (**B**) co-axial electrospinning, (**C**) physical adsorption and (**D**) covalent immobilization.

Blend electrospinning is capable of fabricating hybrid nanofibrous mats through the mixture of different polymer solutions, which also allows the addition of biomacromolecules into these solutions prior to electrospinning. By this means, incorporation of bioactive proteins can be achieved in the form of protein loaded nanofibrous matrices (Figure 4A). So far, a wide variety of proteins and growth factors such as bovine serum albumin (BSA) [183], lysozyme [184], silk gland sericin [185], bone morphogenic protein-2 (BMP-2) [186] and epidermal growth factor (EGF) have reportedly been successfully included into electrospun scaffolds. Because polymer and protein are mixed, the latter tends to be localized in the resulting fiber rather than on its surface. This favors sustained release of the loaded protein, usually over several weeks [187].

Co-axial electrospinning is a dynamic process to obtain nanofibers with core–shell structure and the sheath can protect the core against direct exposure to the environment (Figure 4B). Electric charges are predominantly assembled at the outer surface of the solution jet (shell) during electrospinning, leaving the inner protein solutions (core) free of charge [188]. This is especially interesting for incorporation and delivery of growth factors. This special type of proteins is capable of instructing specific cellular responses like cell growth, proliferation, differentiation, migration and healing, so that the fabricated biomimetic scaffold may induce tissue regeneration through natural cues [20]. This approach has been reported to facilitate homogeneous protein distribution [189]. Through meticulous selection of processing parameters including the feeding rate, interfacial tension and viscoelasticity of the two solutions, the entrapment of core components can be tailored [190]. The protein release from such fibers follows a specific characteristic. Generally, the release from co-axial spinning starts with an initial burst release caused by the quick diffusion of drugs deposited on the fiber surfaces. This is followed by a long period of sustained release, which is the result of synergistic effects of both protein diffusions through the polymer matrix and the slow degradation of the polymer itself.

This flexible technique has been explored for bioactive protein delivery of numerous different types of proteins, including BSA [31], lysozyme [191], platelet-derived growth factor-bb (PDGF-bb) [192], nerve growth factor (NGF) [193] and fibroblast growth factor-2 (FGF-2) [194]. Jiang et al. developed a one-step procedure based on co-axial electrospinning to incorporate BSA and lysozyme to biodegradable core–shell nanofibers (PCL as shell, and protein-containing PEG as core). The release rate could reportedly be controlled by the addition of PEG and water-soluble macromolecules in the sheath material [195]. In another attempt, growth factor containing mesoporous nanocarriers were incorporated into bioactive electrospun hollow fibers to obtain composite scaffolds, which acted as dual growth factor delivery vehicles for tissue engineering applications [196]. Electrospinning under pressurized CO_2_ can also be a useful approach to fabricate polyvinylpyrrolidone (PVP) fibers with hollow interiors. Given the unique properties of PVP (solubility, low toxicity, high complexing ability, adhesive properties, etc.), it is widely used to prepare solid dispersions to improve solubility of poorly water-soluble drugs [197].

Protein can also be used for the shell structure. Ravichandran and colleagues fabricated poly(glycerol sebacate)/fibrinogen (PGS/fibrinogen) core/shell fibers with elastomeric PGS as core, providing suitable mechanical properties and fibrinogen as shell material designed to promote cell–biomaterial interactions. They reported that such core/shell matrices may serve as potential cardiac patch for the regeneration of infarcted myocardium [198]. Further research on suitable materials and solvents in combination with appropriate field strength and balance between electric field strength and feeding rate of solutions will further expand the possibilities of co-axial electrospinning [199].

Aside from co-axial electrospinning, core–shell nanofibers can also be obtained with a conventional electrospinning setup using emulsions of two immiscible solutions [200], such as water-in-oil (W/O) or oil-in-water (O/W) emulsions [201]. Emulsification ameliorates the interaction between biomolecules and organic solvents in which they are suspended. Therefore, emulsion electrospinning has also been considered as effective means of fabricating nanofibrous delivery systems for local and sustained release of biomolecules [202, 203]. Scaffolds fabricated using this method can achieve sustained release profiles lasting for as long as 3 months [204]. The preparation process of suspending the protein solution in the polymer solution by emulsifying can be achieved relatively easy using ultra-sonication or a homogenizer. Tian et al. dissolved vascular endothelial growth factor (VEGF) in a BSA/water solution and dispersed the water phase with the oil phase made of (PLCL in chloroform) to perform emulsion electrospinning. They demonstrated that the resulting nanofibers have a co-axial morphology and the core–shell structured nanofibers offered controlled release of VEGF, possibly useful for cardiac tissue regeneration [205]. This technique has further been proven successful for encapsulation of NGF [206], proteinase K [207], lysozyme [208] and BSA [209].

Physical adsorption is a relatively simple method to immobilize biomolecules to the surface of nanofibers given that proteins are large, amphipathic molecules with intrinsic active surfaces [210]. Thus, they can attach to the scaffolds through non-covalent interactions like Van der Waals force, electrostatic forces, hydrophobic interactions and hydrogen bonding [211]. Basically, the fabricated scaffold is immersed into a biomolecule containing aqueous phase. This is either present in the form of solution or emulsion, and it will be directly coated with protein (Figure 4C). For hydrophobic nanofiber scaffolds, sometimes an additional step of plasma modification is adopted in advance to achieve a more hydrophilic surface, through which the efficiency of the subsequent physical absorption is increased. Notably, ECM protein components such as collagen [212], gelatin [213], laminin [214] and fibronectin [215], which play critical roles in inducing cellular activity during tissue regeneration, can be loaded onto the surface of plasma treated nanofibrous scaffolds. Other functional proteins like heparin [216] and the mussel-inspired protein poly(dopamine) (PDA) [217] have also been reported to be successfully introduced to the nanofibrous scaffold while retaining their own biological effects. A disadvantage of this approach is a relatively uncontrolled release profile.

In contrast to physical adsorption, covalent immobilization uses chemical bonding to immobilize proteins onto the fiber surface, which is more efficient for long-term preservation of these biomacromolecules. A prerequisite for effective protein immobilization is appropriate chemical modification, so that reactive functional groups (e.g. carboxyl, amine or thiol groups) can be introduced into the fiber, facilitating later chemical bonding, where the electrophilic groups on the fiber will react with strong nucleophiles on the protein [218, 219]. In most cases, covalent immobilization is based on N-(3-dimethylaminopropyl)-N′-ethylcarbodiimide hydrochloride/N-hydroxysuccinimide (EDC/NHS) chemistry (Figure 4D). For example, Mahmoudifard et al. demonstrated that anti-staphylococcus enterotoxin B (anti-SEB) can be immobilized onto a nanofibrous electrospun polyethersolfone membrane through EDC/NHS coupling chemistry, and further oxygen plasma treatment effectively increased the amount of antibody immobilization [219]. Many bioactive molecules including ECM proteins or peptides, growth factors as well as functional proteins like heparin or antibodies were therefore entrapped onto the surface of polymer nanofibrous scaffolds for specific tissue engineering purposes [220, 221]. Covalent bonds are stable and irreversible. Thus, they do not desorb uncontrolledly, and the release rate of the immobilized biomolecules can be controlled by the external enzymes [182]. Furthermore, this approach also offers an option to deliver multiple biomolecules, e.g. the sophisticated delivery of more than one growth factor in an appropriate temporo-spatial manner that mimics the key biological cues for tissue regeneration [222].

Last but not least, integrating some separate microcarrier systems such as microspheres into nanofibrous scaffolds is an indirect method to encapsulate growth factors or other biomacromolecules. Hybridization of electrospun nanofibers with protein embedded nano- or microparticles results in an interconnected macroporous structure. This provides effective protection of the incorporated protein against environmental conditions—a mechanism similar to that of co-axial electrospinning [223]. Therefore, protein stability and a prolonged period of protein delivery can be achieved [224]. Moreover, this approach may provide an option for producing multi-drug delivery systems with timed programmed release [225]. For example, various growth factors were first incorporated into PLGA microspheres, and the medicated microspheres were subsequently attached onto a pore wall surface, resulting in high loading efficiency and specific release profiles [226, 227]. Whitehead et al. described a procedure of fabricating PLGA microspheres that contain NGF and directly impregnating the scaffold with these microspheres during the electrospinning process. They demonstrated that protein viability was preserved to stimulate neurite outgrowth after harsh processing conditions like high sheer forces and electrical charges. The system was shown to release protein for over 60 days [228]. Similarly, Sun et al. used positively charged chitosan nanoparticles (CNPs) as carriers to incorporate negatively charged proteins prior to electrospinning. They showed that proteins retained their bioactivity and could be released from polyester nanofibers in a sustained manner for more than 4 weeks without any initial burst [229].

### Combining electrospinning with 3D printing

Electrospinning offers high surface-to-volume ratio but fails to yield a 3D structured material rationally designed with solitary hierarchical architectures as well as mechanical sophistication and conformance. While 3D printing process offers flexibility to fabricate 3D materials with intricate design details but has a major limitation in terms of resolution in micrometers scale. In order to overcome the limitations associated with each of these techniques, reports are now emerging on the combinational use of electrospinning and 3D printing to fabricate a new set of materials with enhanced structural and functional features (Figure 3I-L) [139, 230–232].

A combinational technique was investigated for the fabrication of bi-layered carriers from a blend of PVA and sodium alginate (SA) [233]. The bi-layered carriers were prepared by solvent casting in combination with two surface modification approaches: electrospinning or 3D printing. Kozior et al. describe the results of research on composites manufactured by combining samples prepared with two 3D printing technologies, fused filament fabrication (FFF) and photo-curing of liquid polymer resins (PJM) in combination with electrospinning (ES) technology [234].

Nowadays, there are several different ways to combine the approaches of electrospinning and 3D printing. Fabrication of 3D-printed structures and electrospun nanofibers directly on its surface is one of the most straightforward techniques. 3D printing on tubular or flat nanofibers is the second choice. Alternatively, hybrid scaffolds can be created using 3D printing and electrospinning. The initial layer is made using 3D printing, and then nanofibers are deposited on top using electrospinning. After that, 3D printing and electrospinning techniques are used interchangeably to blend the layers. The electrospun nanofibers sheets can be inserted between the stacks of 3D-printed structures. In addition to these techniques, there is also a combinational approach that involves the use of electrospun nanofibers as ink for the 3D printing process. The formation of scaffolds with adequate mechanical strength is a result of this.

Therefore, the harmonious amalgamation of 3D printing and electrospinning technologies results in mutual benefits, enhancing both material’s plasticity and porosity, as well as improving its mechanical strength. As a result, it renders rational design and manufacturing of materials more appropriate for biomedical applications. Zhao and colleagues utilized 3D bioprinting and electrospinning to create a conductive composite scaffold consisting of polypyrrole and silk fibroin (PPy/SF) [235]. They applied this scaffold to induce electrical stimulation, which was found to be effective in facilitating the treatment of neural injuries and showed great potential in promoting peripheral nerve regeneration. Carranza et al. explored the combination of 3D printing and electrospinning and developed novel scaffolds using natural polymers of chitin and gelatin [236]. Unique ink properties from various biopolymers were utilized to achieve a multilayered scaffold capable of tissue regeneration. The composites of 3D-printed materials and electrospun nanofibers have proven successful in regenerating various other tissues, such as bone, muscle, cartilage, vascular and skin. Also, drug delivery was attempted through 3D-printing with electrospun nanofibers. While most of these approaches focus on the controlled release of small molecules, some bioactive proteins were also included in such scaffolds. Gao et al. introduce a new bi-layered scaffold with nano-/micro-structure, which was created through 3D printing and electrospinning. The scaffold was then modified with 3,4-dihydroxyphenethylamine—epidermal growth factor using bioorthogonal techniques. The protein-containing scaffold demonstrated a significant enhancement in wound healing for rats [237]. Meanwhile, Lee and colleagues demonstrated that gelatin and rhBMP-2-incorporated 3D-printed PLA scaffolds filled with PLGA/Hap electrospun nanofibers can effectively treat segmental bone defects in dog long bones [238]. Taken together, the combination of 3D printing and electrospinning technologies offers significant potential in the field of biological tissue regeneration.

## Applications in wound healing

A wound is the destruction of the integrity of the skin or mucous tissue of an internal organ, leading to the exposure of the internal structure. Wound repair, on the other hand, involves the physiological response of the body to this tissue damage and the restoration of tissue integrity. Wound healing is a highly ordered biological process that involves early coagulation and hemostasis, an inflammatory response, cell proliferation and migration, and later remodeling and regeneration of new tissue [239]. Unfortunately, wound healing typically involves fibrosis and scar tissue formation, with scar hyperplasia resulting in skin itching, pain, disfigurement, impaired perception, increased infection risk and loss of physiological functions. These consequences significantly impact patients’ daily lives. Accordingly, researchers and clinicians have focused on developing novel technical methods for wound regeneration and repair. While materials prepared through the process of electrospinning have demonstrated significant success and exhibit a promising potential for the treatment of wounds.

### Electrospun wound dressing

**Figure 5.**
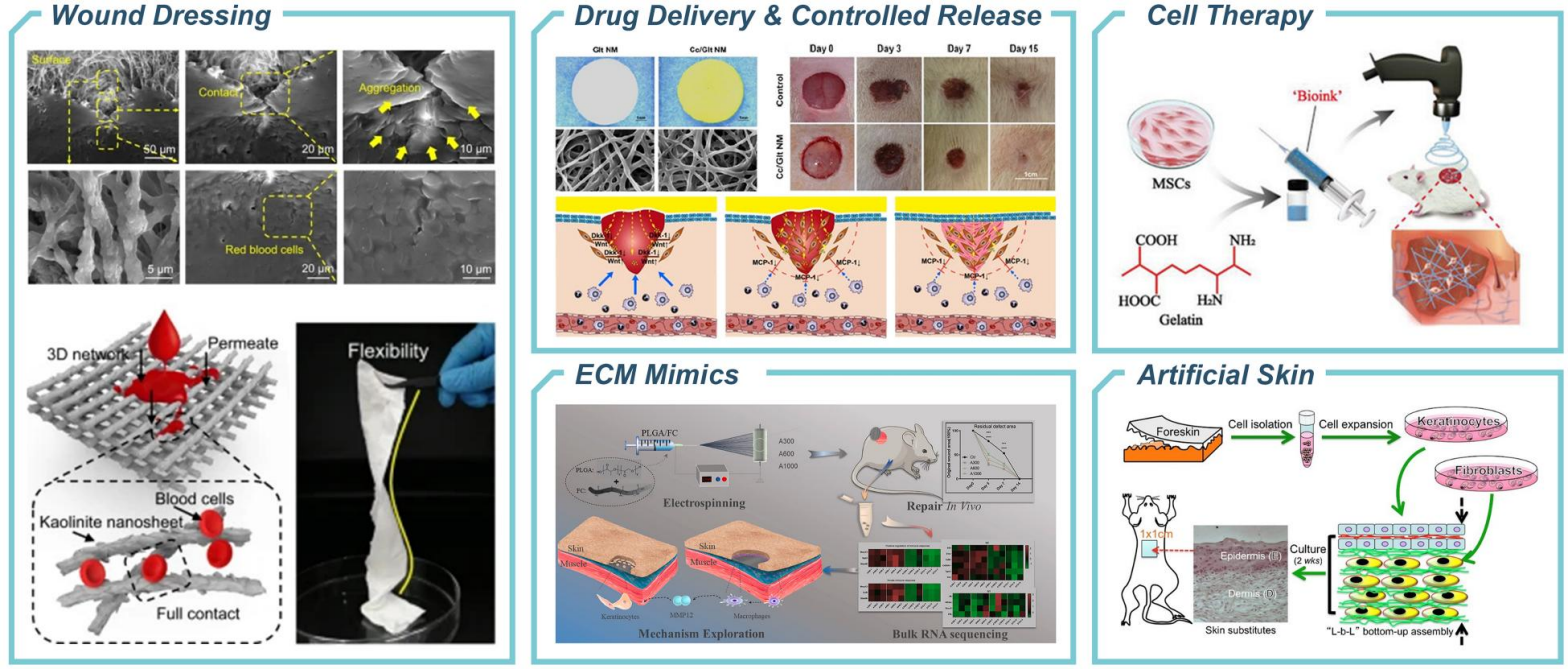
Main mechanisms and effective approaches of materials produced *via* electrospinning for wound repair applications. Reproduced with permission from [89, 246–249]. Copyright 2021 Springer Nature, 2017 Springer Nature, 2022 Elsevier, 2023 John Wiley and Sons and 2015 Elsevier.

The most direct use of electrospinning in wound treatment involves the production of nanofibrous mats. The combination of diverse constituent fibers allows the mat to demonstrate either efficient water absorption or waterproofness, in addition to superior mechanical elasticity and flexibility [240–244]. Meanwhile, the porous nature of these mats enables their application as water resistant, breathable, moisturizing and rapidly hemostatic dressings for sealing wounds during the healing process. Yue et al. utilized ethanol-soluble polyurethane (EPU) and fluorinated polyurethane (FPU) as polymer carriers, deploying a bespoke electrospinning contraption to craft a thymol-loaded nanofiber membrane. The results show that the membrane features breathability and waterproofing properties, as well as excellent antibacterial activity [245]. Zhang and colleagues developed nanoclay-organic self-supported membranes through electrospinning [246]. The sheet-like kaolinite was uniformly dispersed in and partly exposed on PVP fibers, which aided in stabilizing the framework and regulating the spontaneous shrinkage and degradation of the PVP fibers. This structure design also imbues the membrane surface with high hydrophilicity, potentially inducing blood coagulation by quickly aggregating its constituents, activating platelets and initiating the intrinsic coagulation pathway (Figure 5 left panel). Attributed to its enriched hemostatic functional sites, robust framework and hydrophilic surface, the newly developed membrane exhibits improved comprehensive performance in treating rat tail amputation as well as liver and spleen wounds. This marks a promising blueprint for advancing electrospun wound dressings.

### Drug delivery and controlled release

Electrospun fibers are highly sought after for drug loading and delivery applications due to their unique combination of properties. These fibers have diameters ranging from micro- to nanoscale, resulting in a significantly increased surface area compared to bulk materials. This expansive surface area allows for efficient drug adsorption and encapsulation. The porosity of electrospun fibers enables the incorporation of drugs within their network of pores, allowing for controlled drug release kinetics and targeted delivery to the desired site of action. Furthermore, the electrospinning process allows for precise control over the morphology of the resulting fibers, enabling the creation of customized fiber architectures to optimize drug loading, release profiles and interaction with biological systems. This platform is positioned as a leading option for developing versatile drug delivery systems in wound healing.

#### Antimicrobial elements release

In addition to wound closure and hemostasis, incorporating antimicrobial properties is a valuable addition to wound dressings. Thus, introducing antibacterial elements into electrospun fibers, including inorganic nanomaterials, small molecules (such as antibiotics and gasses) and biological macromolecules with antimicrobial properties, can grant the resultant membrane effective antibiotic capabilities [250–255]. Xie et al. confidently developed a film with broad-spectrum antibacterial activity for the treatment of infected wounds by combining electrospun nanofibrous scaffolds with 4,6-diamino-2-pyrimidinethiol-modified gold nanoclusters [256]. In a similar vein, Guex and colleagues diplomatically proposed a potential alternative for treating infected wounds by designing a bifunctional peptide that combines an antimicrobial peptide and a cellulose binding peptide, and immobilizing it on electrospun fibers of plant-derived cellulose [257].

Simultaneously, the degradation products of the fiber components can also exhibit antibacterial properties, thereby enabling the dressing to curb bacterial growth during wound treatment. Studies have shown that dressings imbued with antimicrobial capabilities significantly enhance therapeutic outcomes in the treatment of wounds, particularly those chronic wounds with prolonged infections or heightened infection risks [258].

#### Delivery of bioactive molecules promoting wound healing

Electrospun fibers can contain antimicrobial ingredients, as well as other bioactive molecules that aid wound repair. These molecules contribute to immunoregulation, promoting cell proliferation and migration, and facilitating angiogenesis and tissue remodeling [259, 260]. Dai et al. presented curcumin/gelatin-blended nanofibrous mats fabricated by electrospinning [89]. These mats were created to enhance the bioavailability of the hydrophobic curcumin, which was formulated as an amorphous nanosolid dispersion and sustained release from gelatin-based biomimetic mats. The drug-loaded mat improved the regenerative process in acute rat wounds by increasing the mobilization of wound site fibroblasts and inhibiting the inflammatory response (Figure 5 upper middle panel). Furthermore, the combination of the curcumin/gelatin-blended nanofibrous mat’s effective cancer suppressor effect suggests it could be an affordable, secure and efficient substitute for eliminating residual cancer cells and promoting surgical wound healing after tumor resection [261].

The controlled release of medication loaded in electrospun fibers can occur *via* external stimuli including light, heat and fiber degradation, in addition to unrestricted diffusion from the fiber into the environment [262, 263]. Furthermore, electrospun fibers may be customized to include active structural components that function as catalytic substrates for specific enzymes expressed or activated within wound areas, such as matrix metalloproteinases. In an environment with enzyme activity, such fibers disperse the enclosed drug upon breakdown. Moreover, some of the fabricated fibers have the capacity to generate physical stimulation, such as an electrical field, which can facilitate cell migration and proliferation, thereby promoting wound healing [264, 265].

#### Gene editing vector

Moreover, advancements in gene therapy and gene editing technologies have resulted in the exposure of *in situ* gene editing and gene therapy of lesions by incorporating and liberating siRNA and CRISPR-Cas systems through electrospun fibers. Kobsa et al. synthesized electrospun scaffolds composed of a blend of degradable polymers and plasmids encoding keratinocyte growth factor [266]. When applied to full-thickness wounds in mice, these scaffolds significantly improved the rate of wound re-epithelialization, keratinocyte proliferation and granulation response, demonstrating their effectiveness in enhancing wound healing. Li and colleagues used polyplexes of a plasmid encoding basic fibroblast growth factor with poly(ethylene imine) to create electrospun fibers with a core-sheath structure [267]. They applied these fibers to the dorsal wound of diabetic rats and found that the gradual release of pbFGF polyplexes resulted in a significantly higher wound recovery rate, collagen deposition and maturation, complete re-epithelialization and skin appendage regeneration. These studies demonstrate that combining electrospinning with gene techniques can address the challenges of complex targeted administration. The potential implementation of this approach in wound management is currently under evaluation and receiving greater attention.

#### Potential challenges

Electrospinning is a promising technique for drug delivery and controlled release in wound healing applications. However, there are still some challenges and limitations that need to be addressed. The electrospinning process frequently utilizes organic solvents or UV radiation, which can negatively impact drug stability. Additionally, the high shear forces inherent to the technique may denature certain drugs. Meticulous selection of compatible drug–polymer combinations is necessary to mitigate drug degradation. Alternative solvents or processing conditions may also need to be explored.

Meanwhile, one significant hurdle in controlled release is the initial burst release. Conventional blending methods often result in a large portion of the drug being released immediately upon application of the electrospun fibrous and porous structures. This can lead to supra-physiological drug concentrations at the wound site, potentially causing cytotoxicity and reducing therapeutic efficacy. Moreover, wound healing is a complex, multi-cellular and multi-factorial process that involves multiple stages occurring at different times and positions. Precise control of the release of active molecules that affect specific targets is necessary during wound treatment. However, tailoring the release rate and duration of therapeutic agents from electrospun nanofibers remains a challenge. The release profile is influenced by various factors, such as the physicochemical properties of the drug and the nanocarrier (e.g. fiber diameter, porosity), as well as the processing parameters used during electrospinning (e.g. applied voltage, flow rate). Achieving a sustained and controlled release profile requires a thorough understanding of the interplay between these variables.

Last but not least, while electrospinning offers a versatile platform for drug delivery, its current application is largely confined to the laboratory setting. Scaling up production for clinical use presents significant challenges. These include maintaining process consistency at larger scales, ensuring cost-effectiveness for large-batch production and implementing robust quality control measures.

As previously stated, researchers are actively addressing these limitations through the development of novel electrospinning techniques such as co-axial electrospinning and emulsion electrospinning. Furthermore, exploring alternative biocompatible polymers, optimizing processing conditions and developing condition-responsive release mechanisms offer promising avenues for improving drug delivery and controlled release in wound healing applications.

### Regenerative scaffolds and ECM mimics

Numerous crucial steps in the wound healing process rely on the wound microenvironment. For instance, the adsorption substrates of fibroblasts and keratinocytes impact their migration and proliferation. Therefore, the ECM, which has a vital role in the wound microenvironment, has long been a key focus in wound healing [268]. Electrospun nanofibrous membranes exhibit adjustable hardness, surface roughness and fiber diameter, as well as a favorable charge and hydrophobicity. The controlled creation of specific surface morphologies and patterns allows to effectively mimick natural ECM. As such, it serves as a mechanically and physically beneficial scaffold to aid wound cell activation and migration [263, 269, 270]. Sun et al. reported on an electrospun nanofibrous scaffold that mimics the basketweave-like pattern of collagen fibrils in native skin. The scaffold stimulated a range of cellular responses in fibroblasts, leading to alterations in their morphology and gene expression associated with wound healing. Furthermore, it aided the resolution of inflammation, promoted fibroblast and keratinocyte migration and facilitated angiogenesis, thereby contributing to the restoration of wounds in diabetic rats [271].

ECM mimics produced *via* electrospinning can replicate the physical properties that provide natural ECM while also being functionalized through surface modification with bioactive molecules or ECM components. This enables biological activity and functions of natural ECM [272, 273]. Burdick and colleagues introduced an innovative method that merges electrospinning and photon patterning to deliberately and locally modify the electrospun fiber surface with RGD, a peptide motif that encourages cell adhesion to the ECM [274]. Spatially patterned RGD impacted cell adhesion and morphology, enhancing cell density, elongation and area in regions with RGD, while preserving cell orientation with nanofiber alignment. Thus, the scaffold can exert independent control over the physical and biochemical properties, resulting in a synergistic effect on cells, leading to the formation of more complex cell–material interactions. Consequently, this facilitates more precise regulation of cell behavior and enables a closer imitation of the natural ECM, from physical structure to biological function. Qian et al. functionalized electrospun silk fiber membranes through surface modification with PDA and encapsulated leptin liposomes [275]. As a result, the functionalized membrane exhibited a marked enhancement in tensile strength, hydrophilicity, fibroblast adhesion and angiogenesis. The membrane additionally accelerated the recovery of rabbits’ oral mucosa wounds.

Electrospun fibrous scaffolds not only enable the regulation of cellular responses directly associated with wound healing, but also can stimulate cells to express and secrete bioactive molecules to optimize the wound microenvironment and accelerate the healing process. A prevalent example of this concept involves the implantation of a scaffold regulating the immune environment of a wound. The electrospun scaffold developed by the Qu & Man group stands out as a remarkable achievement. This scaffold employs PLGA and fish collagen as basic materials and spatially mimics the ECM structure of the skin. It optimizes fiber diameter and orientation to promote cell proliferation and spreading, which aid in skin wound healing through vascularization and epithelization (Figure 5 lower middle panel). Additionally, this scaffold contributes to immature hair follicle regeneration. To enhance the comprehension of the underlying mechanism, they utilized a combination of single-cell RNA sequencing and spatial transcriptomics to examine the full-thickness skin located in the wound vicinity following the insertion of the scaffold. The findings reveal how scaffold placement regulates T cells and other lymphocytes, which in turn influence the conduct of fibroblasts and keratinocytes *via* the secretion of inflammatory factors and the modulation of the immune environment [248, 276, 277]. The consequence of this is the promotion of wound regenerative repair.

### Cell printing and cell therapy

Some injuries, particularly those with extensive tissue loss, suffer from insufficient cells during the repair process, leading to delays or hurdles in the healing process [278]. Cell therapy seeks to transplant human cells to repair or replace damaged tissues or cells, making it an ideal strategy to address such difficulties. Nevertheless, the biggest challenge in cell therapy has always been accurately delivering cells with therapeutic functions to the targeted region while sustaining their activity [249]. Therapeutic cells can be integrated into electrospun scaffolds and topically implanted into the target treatment area. Electrospun scaffolds is an effective method for overcoming transportation difficulties and enhancing the efficacy of cell therapy. In 2006, Townsend-Nicholson and Jayasinghe were the first to enclose astrocytoma (1321 N1) cells in electrospun fibers, leading the way for the use of electrospinning technology in cell therapy [279]. Xie and colleagues showed that 3D radially and vertically aligned nanofiber scaffolds could be used to transplant bone marrow mesenchymal stem cells (BMSCs). The 3D scaffolds containing BMSCs promote formation of granulation tissue, angiogenesis and collagen deposition while redirecting immune responses toward the pro-regenerative direction, leading to a marked improvement in managing diabetic wounds [280].

Challenges of using electrospun fibrous scaffold-assisted cell therapy includes the removal of toxic solvents from the scaffolds, adequate cell infiltration, uniform cell distribution, achieving encapsulation and *in vitro* cell cultivation [281]. Printing living cells with substrate materials during fiber fabrication can improve cell distribution and infiltration, which helps functional connectivity among cells and accelerates the regeneration and repair of the tissues, despite possible cell inactivations or death [282]. Wen et al. demonstrated direct placement of bioactive fish gelatin fibers and BMSCs onto wound sites *via in situ* cell electrospinning, which can maintain cell viability above 90%. The treatment has been demonstrated to improve the restructuring of the ECM by boosting the deposition of collagen, stimulate the growth of new blood vessels by increasing the expression of vascular endothelial growth factor, and significantly decreasing the expression of interleukins in the process of wound healing (Figure 5 upper right panel). As a result, wound healing is accelerated and scarring is reduced [249].

### Artificial skin

Autologous tissue transplantation and allogeneic tissue transplantation are commonly employed methods for the regeneration and repair of skin tissue in the treatment of large skin injuries and refractory wounds [283]. While these methods are considered the gold standard, they have limitations including immune rejection by patients, secondary trauma, limited practicality and high cost [284]. Creating artificial skin *in vitro* that closely resembles natural skin has been a longstanding objective of tissue engineering for use in flap transplant surgery.

The skin is a complex, multilayered structure made up of epidermal cells, fibroblasts and vascular endothelial cells. Most current artificial skin products are based on this understanding. To create an asymmetric 3D skin construct, Correia and colleagues employed electrospinning and 3D bioprinting techniques. They used a blend of PCL and silk sericin in an electrospun upper layer that closely replicates the characteristics of the epidermis. The dermis-like layer was created by printing a hydrogel made of chitosan and SA. This structure has various qualities including morphology, porosity, mechanical properties, wettability, antimicrobial effects and non-toxicity, which make it a suitable option for skin replacement during the healing process [285].

The electrospinning technique as described in Section ‘Cell printing and cell therapy’ can produce fibrous scaffolds that can be used for *in vitro* cell culture to create tissue blocks that exhibit autonomous growth, and complex physiological processes, closely resembling real skin [286]. The adhesion and growth ability of primary cells and somatic cells, which were induced from stem cells, were successfully demonstrated on the electrospun scaffolds [287–289]. The effectiveness of a nanofibrous scaffold created through electrospinning and cultured with a mixture of dermal fibroblasts and keratinocytes in repairing full-thickness wounds was investigated. Park and colleagues reported the fabrication of 3D nanofibrous structures using cold-plate electrospinning. These structures were subsequently co-cultured with human dermal fibroblasts and keratinocytes to create a skin substitute [290]. Additionally, both Xie’s and MacNeil’s research teams included minced skin grafts between the electrospun fibrous scaffolds to enhance wound healing following implantation [291, 292]. Furthermore, Wang and colleagues employed a layer-by-layer approach to create skin tissue utilizing electrospun PCL/collagen nanofibers and foreskin cells, specifically fibroblasts and keratinocytes (Figure 5 lower right panel) [247, 293]. In a similar vein, Khandaker and colleagues cultured human dermal fibroblasts in a blend of collagen gel and electrospun PCL membrane to create dermal equivalent grafts. Keratinocytes were added on top to allow for epidermal growth. After either *in vitro* culturing or *in vivo* implantation, both of these construct types exhibited dermal and epidermal compartments’ corresponding layers and a potential for regenerative repair of wounds [294]. Although the current electrospinning-based artificial skin lags slightly behind the hydrogel layered culture method in terms of structural and functional integrity, the addition of electrospinning enhances mechanical strength and enables more precise and controllable manufacturing of structures. This advancement presents a promising future for perfecting artificial skin fabrication.

### Clinical applications

The aforementioned laboratory research achievements provide a robust foundation for the utilization of electrospinning in actual clinical wound treatments. A number of clinical trials have been conducted in this field. While not all cases have yielded positive outcomes, a significant number of successful clinical trials have demonstrated the efficacy of electrospun membranes in various wound healing scenarios. In a comparative study, Husain et al. evaluated the therapeutic efficacy of a commercial product comprising a synthetic electrospun fiber matrix in the treatment of diabetic foot ulcers, contrasting it with the use of foam or alginate dressings. The findings indicate that, following a 12-week course of treatment, the proportion of 100% re-epithelialization in the wound area and wound closure in patients receiving treatment with an electrospun fiber matrix was significantly higher than in the control group [295]. In contrast, López-Jaramillo et al. initiated an investigation utilizing the meglumine antimoniate patch for the treatment of wounds. The electrospun fiber mat was observed to significantly promote re-epithelialization in the wound, yet it also affected the mucous membranes, ultimately resulting in the termination of the trial [296]. Another promising outcome is documented by Arenbergerova et al. The administration of a commercial polyurethane nanofibrous mesh to 162 patients with chronic leg ulcers led to a reduction in wound size among 35% of trial participants, while 71% of trial participants reported a reduction in pain [297].

In conclusion, the number of electrospun fiber materials employed in wound healing trials is considerably smaller than the number of such materials documented in academic literature. Furthermore, the number of materials that successfully complete the trial and are subsequently incorporated into clinical therapy is even smaller. This is a common phenomenon for most biomaterials during their translation to clinical applications, given that the human body is a much more complex system than cell cultures, mice or rats. Nevertheless, the considerable quantity of material produced in laboratory settings offers a substantial pool of potential candidates for consideration by clinical staff. The identification of more and superior materials will be enhanced by the publication of a greater number of materials in scientific journals.

## Advantages and challenges

Rapid advances in the field of tissue regeneration have created new challenges in how to induce natural physiological responses to repair damaged tissues. It has thus become a promising field for individualized design of bioactive ‘smart’ scaffolds capable of providing physical support not only for cells but also for functional biomacromolecules, especially growth factors. In this regard electrospinning has proved to be an outstanding technique to meet these needs by fabricating nanofibrous matrices with tailored ECM-mimicking architectures and medicinal properties. Moreover, the interconnectivity, the 3D porous structure, the enormous surface area, as well as the wide spectrum of biodegradable materials allows for encapsulation and delivery of therapeutic proteins. For example, water-soluble therapeutic proteins encapsulated in biodegradable electrospun fibrous matrices have reportedly superior controlled release properties in terms of loading efficiency/capacity, mild preparation conditions and relatively steady release characteristics [195]. Delivery systems such as micro/nanoparticles, although proven to be effective, can only act as biomacromolecule carriers rather than supportive scaffolds. Likewise, it is often difficult to keep them localized in the desired tissue area. Hence, electrospinning can provide the opportunity to integrate protein embedded micro/nanocarriers into nanofiber matrices for improved biological properties (e.g. local release of growth factors, and promoting the accuracy of tissue development both *in vitro* and *in vivo*).

Despite recent advances in electrospinning for preparation of biomimetic scaffolds, many challenges still exist. Although electrospinning has been widely accepted as a relatively simple process, the principles behind this technique turn out to be complicated, covering a wide range of basic scientific fields such as electro-statistics, fluid-rheology and macromolecular chemistry. More studies are needed to fully understand the underlying mechanisms for improving control over morphology, size and other architectural parameters. Furthermore, electrospun matrices are comprised of closely-packed nanofibers with macro/nanometer diameter range sizes. It is reported that there is a positive correlation between fiber diameter and the average pore radius [298]. Therefore, electrospun nanofibrous scaffolds may only possess superficial porous structures, in which case cellular infiltration and tissue ingrowth are largely hindered. Many attempts have been made to reduce the packing density and enlarge the pore diameter of fibrous materials, including the use of sacrificial components [299], novel configurations of the collector [300] or synchronizing cell seeding with fiber formation during electrospinning [301]. Nonetheless, more effort is still required to ensure realistic porous structures for electrospun fibers. A bigger challenge regarding the encapsulation and delivery of functional proteins/growth factors is preservation of their bioactivity. Despite significant progresses for safe incorporation of biomacromolecules by means of blend/co-axial/emulsion electrospinning, they are still susceptible to denaturation before they can be released. Protein unfolding, de/rehydration and exposure to harsh processing conditions (e.g. high electrical charges, organic solvents, acidic environments caused by the degradation products) can cause denaturation and subsequent loss of functionality [302]. Even a dry environment is not necessarily favorable for the stability of proteins at physiological temperatures [301]. Further studies are therefore needed to address this issue. For example, a novel but largely unexplored strategy of encapsulating protein crystals into electrospun matrices may provide new options for improving solvent resistance [303]. Accurate control over release profile is another critical challenge in maintaining desirable temporo-spatial concentration of bioactive proteins [301]. An ideal electrospun scaffold should exhibit a release profile within the time-frame of tissue regeneration. Such an efficiency in delivery, however, is often limited by fragile structure and complicated monitoring of therapeutic proteins. In view of this, co-axial electrospinning may be one of the answers to fine-tune processing parameters for preserving protein activity and controlling its release rate. Likewise, a consistent core–shell structure throughout the fiber length may be valuable for an improved release profile. It should be noted that in a natural process of tissue regeneration, cells usually interact with soluble growth factors in a gradient concentration. It is therefore very important for future studies to emphasize fabricating biomimetic scaffolds with simulated gradient of biomacromolecules in order to provide favorable signaling. Last but not the least, simultaneous delivery of multiple growth factors to a selected region from the same electrospun scaffold is shown to be more advantageous compared to those capable of releasing a single growth factor [301]. Further investigations are required to optimize this strategy.

In conclusion, electrospinning has emerged as a remarkably simple and versatile technique for constructing ECM-mimicking scaffolds. With careful selection of proper materials, as well as processing techniques and parameters, it is possible to tailor nanofibrous scaffolds with adjusted biochemical and topographical cues for improved tissue regeneration. Although impressive progress has been made in applying electrospun biomimetic scaffolds for regenerative repair, challenges still exist. A better understanding of the underlying principles of electrospinning, and further improvement of structural, chemical, biological and mechanical properties of electrospun scaffolds may prove to be groundbreaking for therapeutic applications in the near future.

## Funding

This work was supported by the National Natural Science Foundation of China (82072174), National Key R&D Program of China (No. 2020YFA0908100) and CAS Key Laboratory of Interfacial Physics and Technology (CASKL-IPT1702).

*Conflicts of interest statement*. The authors declare no conflict of interest.
